# Deciphering the Role of PIG1 and DHN-Melanin in *Scedosporium apiospermum* Conidia

**DOI:** 10.3390/jof9020134

**Published:** 2023-01-18

**Authors:** Hélène Guegan, Wilfried Poirier, Kevin Ravenel, Sarah Dion, Aymeric Delabarre, Dimitri Desvillechabrol, Xavier Pinson, Odile Sergent, Isabelle Gallais, Jean-Pierre Gangneux, Sandrine Giraud, Amandine Gastebois

**Affiliations:** 1 CHU Rennes, INSERM, EHESP, IRSET (Institut de Recherche en Santé Environnement et Travail)—UMR_S 1085, 35000 Rennes, France; 2 University of Angers, University of Brest, IRF, SFR ICAT, 49000 Angers, France; 3 INSERM, EHESP, IRSET (Institut de Recherche en Santé Environnement et Travail)—UMR_S 1085, 35000 Rennes, France; 4 Institut Pasteur, Université Paris Cité, Plate-Forme Technologique Biomics, 75015 Paris, France; 5 Institut Pasteur, Université Paris Cité, Bioinformatics and Biostatistics Hub, 75015 Paris, France; 6 CNRS, INSERM, Biosit UAR 3480 US_S 018, MRic Core Facility, 35000 Rennes, France

**Keywords:** *Scedosporium apiospermum*, DHN-melanin, cell wall, PIG1, CRISPR-Cas9, RNA-seq

## Abstract

*Scedosporium apiospermum* is a saprophytic filamentous fungus involved in human infections, of which the virulence factors that contribute to pathogenesis are still poorly characterized. In particular, little is known about the specific role of dihydroxynaphtalene (DHN)-melanin, located on the external layer of the conidia cell wall. We previously identified a transcription factor, PIG1, which may be involved in DHN-melanin biosynthesis. To elucidate the role of PIG1 and DHN-melanin in *S. apiospermum*, a CRISPR-Cas9-mediated *PIG1* deletion was carried out from two parental strains to evaluate its impact on melanin biosynthesis, conidia cell-wall assembly, and resistance to stress, including the ability to survive macrophage engulfment. Δ*PIG1* mutants did not produce melanin and showed a disorganized and thinner cell wall, resulting in a lower survival rate when exposed to oxidizing conditions, or high temperature. The absence of melanin increased the exposure of antigenic patterns on the conidia surface. PIG1 regulates the melanization of *S. apiospermum* conidia, and is involved in the survival to environmental injuries and to the host immune response, that might participate in virulence. Moreover, a transcriptomic analysis was performed to explain the observed aberrant septate conidia morphology and found differentially expressed genes, underlining the pleiotropic function of PIG1.

## 1. Introduction

*Scedosporium* species are filamentous fungi widely distributed throughout the environment, especially in soil, where they live as saprophytes. However, they can also behave as opportunistic pathogens*. Scedosporium* sp. are involved in a large range of human diseases, from chronic localized infections to immuno-allergic diseases in immunocompetent hosts and severe invasive infections in immunocompromised patients [1]. *Scedosporium* is also the second most prevalent mold after *Aspergillus* involved in airway colonization of cystic fibrosis (CF) patients, with a prevalence ranging from 3 to 25% depending on the geographical area and technical procedures used for mycological culture [2,3,4]. In addition, there has been an increasing number of reports on the involvement of *Scedosporium*/*Lomentospora* in invasive life-threatening infections in individuals with underlying immune-system impairment, including those with hematological malignancies and bone marrow or solid organ transplants. [5,6]. The observed poor outcome is also related to the frequent resistance to systemic mold-active antifungals available to treat such infections [7]. 

Disseminated infections result from fungal escape from the early innate immune response, i.e., the resistance of infectious propagules (conidia) against oxidative killing by macrophages and neutrophils [8,9]. There is a growing interest in deciphering the structural properties of the fungal cell wall, which confers high resistance against exogenous injuries. The conidia cell wall of most fungal pathogens has been shown to be covered with a dark pigment, consisting of melanins, such as those particularly well-described in *Aspergillus fumigatus* [10] and *Cryptococcus neoformans* [11]. Among filamentous fungi, the most highly conserved structure is dihydroxynaphtalene (DHN)-melanin, biosynthesized through the pentaketide pathway, initiating from endogenous acetylCoA/malonylCoA, which is converted into 1,3,6,8-tetrahydroxynaphtalene (THN) by polyketide synthase (PKS). Then, 1,3,6,8- THN is reduced by a specific reductase to produce scytalone, which is, in turn, enzymatically dehydrated to 1,3,8-trihydroxynaphtalene. Vermelone is produced through a second reduction step and finally dehydrated to form 1,8-dihydroxynaphtalene, which is dimerized and polymerized to form DHN-melanin [12].

DHN-melanin is often mentioned as ‘fungal armor’, due to the ability of the polymer to protect microorganisms against a broad range of toxic insults. This highly hydrophobic and stable structure has been shown to provide conidia with various biological properties, including paramagnetism, the absorption of radiation, and protection against high temperatures and oxidative stress. Concerning the interaction with host cells, a growing number of reports have attributed a crucial role of melanins in fungal virulence both in plant and human pathogen models [13,14]. In particular, DHN-melanin has been extensively studied in *A. fumigatus* [10,15] and several phaeohyphomycetes involved in chronic infections, such as *Exophiala* [16] and *Fonsecaea* [17], using various melanin-deficient mutant-based approaches. For example, the removal of the melanin layer through the deletion of *PKS* was shown to enhance the inflammatory response of *A. fumigatus* conidia-infected human peripheral blood mononuclear cells (PBMCs), highlighting the melanin property of masking surface pathogen-associated molecular patterns (PAMPs) [18]. The killing of internalized melanized *A. fumigatus* conidia was also delayed relative to killing of melanin-deficient conidia via the inhibition of phagolysosomal fusion in a macrophage model, supporting the protective role of DHN-melanin during the host-conidia interaction [19]. In vivo, the virulence of albino *A. fumigatus* strains in a murine model was also attenuated [20]. In addition, the melanin conidial coating has been shown to hamper the in vitro efficacy of certain antifungal drugs, such as amphotericin B, as shown, for example, in *C. neoformans*, *Histoplasma capsulatum*, and *Exophiala dermatitidis* [21,22,23]. 

The structure of the *Scedosporium* conidia cell wall has only been superficially explored. As discussed in the recent review by Rollin-Pinheiro et al. [24], a few reports have recently emerged that highlight the relevance of certain specific cell-wall components in our understanding of host-pathogen interactions, especially carbohydrates. For example, peptidoramnomannans, α-glucans, and glycosylsphingolipids have been shown to play a crucial role in triggering innate immune responses and are key-elements for fungal growth and virulence [25,26,27].

**Figure 1:**
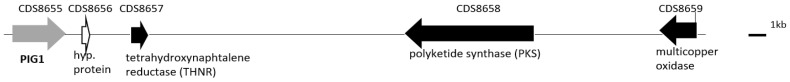
Gene cluster of melanin biosynthesis in *S. apiospermum* (36.1 kb), from the published *S. apiospermum* genome [30]. The scytalone deshydratase encoding gene is located out of the cluster.

Although a very small number of reports have focused on the related genus *Lomentospora* [28,29], there is still no data on the role of DHN-melanin in *S. apiospermum* conidia. By analogy with other filamentous fungi, especially *A. fumigatus*, the biosynthesis of DHN-melanin in *S. apiospermum* may result from the activation of a complex enzyme cascade involving four genes identified in the *S. apiospermum* genome (Figure 1), fully sequenced by our group [30]. Interestingly, three of these genes are organized in a cluster that is thought to be under the control of the upstream transcription factor PIG1, first described in *Magnaporthe grisea*, responsible for rice blast [31]. Many studies dealing with phytopathogenic fungi have supported the importance of genes orthologous to *PIG1* as key regulators of melanin biosynthesis, as demonstrated, for example, in the Δ*CMR1* mutant of *Cochliobolus heterostrophus* [32] and *Colletotrichum gleosporioides* [13] and the Δ*AMR1* mutant of *Alternaria brassicola* [14]. However, different conclusions were drawn concerning the role of these transcription factors in the melanin-related virulence of these fungi, probably because of the diversity of fungal structures involved in plant invasion (i.e., appressoria, microsclerotia, conidia) and differences between the plant models. To date, the contribution of *PIG1* orthologs in melanin biosynthesis and virulence in a human fungal pathogen has never been explored. 

In this context, we aimed to assess the functions of PIG1 and DHN-melanin in *S. apiospermum* conidia using three complementary approaches applied to *PIG1*-deleted albino strains vs. parental *S. apiospermum* strains: (i) phenotypic characterization, (ii) transcriptomic analysis, and (iii) the evaluation of the macrophage response. 

## 2. Materials and Methods

### 2.1. Strains and Culture Conditions

Two different parental strains were used in this study:

- The *Scedosporium apiospermum* wild-type strain deposited at the BCCM/IHEM culture collection (Brussels, Belgium) under the accession number IHEM 14462 (WT 14462) was isolated in 1998 from a sputum sample of a CF patient. The genome of this strain has been sequenced and annotated [30];
- A non-homologous end joining- deficient strain (Δ*KU70*) previously obtained from the WT 14462 strain [33].

All strains were conserved frozen at −80 °C and cultured at 37 °C. Fungi were routinely maintained by passage on supplemented potato dextrose agar plus 0.5% chloramphenicol supplemented with phleomycin (20 µg/mL) for the culture of the Δ*KU70* strains. Hygromycin B (50 µg/mL) was also added to the culture medium of the PIG1-disrupted strains to maintain the selection pressure. Moreover, the WT 14462 strain was cultured in Potato dextrose agar (PDA) supplemented with 50 µg/mL tricyclazole (TRC, PESTANAL^®^, Sigma-Aldrich) to obtain a melanin-deficient control (WT-TRC). 

All experiments were performed with conidia harvested from nine-day cultures by scraping. The conidia were then filtered through sterilized Miracloth (pore size 22–25 µm) for hyphae removal and washed once in sterile water. Conidia were counted using a LUNA™ automated particle counter (Logos Biosystems, Villeneuve d’Ascq, France). 

### 2.2. Disruption of the PIG1 Gene

The alignment of *S. apiospermum* PIG1 protein sequence with homologous sequences of phytopathogen fungi [31] let to adjust the nucleotide sequence of PIG1- encoding gene (SAPIO_CDS8655), previously annotated [30]. The 2793 bp gene encoding PIG1 was disrupted using CRISPR-Cas9 technology to induce a double-strand break at the 5′ end of the *PIG1* gene using a specific crRNA protospacer (sequence in Table S1). After preparation of the gRNA by mixing crRNA and the universal tracrRNA (Table S1) in equimolar amounts at a final concentration of 33 μM (95 °C for 5 min and then RT for 15 min), RNP complex formation was performed according to the method developed by Abdallah et al. for *A. fumigatus* [34]. 

The repair cassette was obtained by amplification of the hygromycin-resistance gene (hygromycin B transferase—HYGR) from the plasmid pCB1636 using M13 primers flanked by an 80-bp sequence homologous to that of the extremities of the target gene. PCR amplification was performed using Q5 high-fidelity DNA polymerase. 

Transformation was performed as described previously [35] on 10^7^ protoplasts from the two strains of *S. apiospermum* (WT 14462 and Δ*KU70*) with 5 μg of repair cassette and 13.25 μL of the RNP complex (manuscript in preparation). Cultures incubated at 37 °C were checked daily for disrupted upward growth in the selection marker layer. Monosporic isolates of disruptants were then subcultured on YPDA (yeast potato dextrose agar) supplemented with 20 μg/mL phleomycin and/or 50 μg/mL hygromycin B. Genomic DNA was extracted using phenol/chloroform.

The genotype was analyzed and validated using two different methods: (i) PCR amplification with primers designed against the upstream and downstream regions of the insertion and sequencing of the amplicons and (ii) Southern blotting, as previously described [35].

### 2.3. Melanin Detection by Electronic Paramagnetic Resonance (EPR)

A 5 × 10^7^/mL conidial suspension was prepared in sterile water and 300 µL transferred to a quartz EPR tube. EPR examination was performed at 100 K using an Elexsys EPR spectrometer (Bruker, Wissembourg, France) with a 3330 G center field, 2 mW microwave power, 9.377 GHz microwave frequency, 10 G modulation amplitude, and 100 kHz modulation frequency. Synthetic melanin (ref M8631, Sigma Aldrich, Saint-Quentin-Fallavier, France) was used as a standard for melanin quantification. Experiments were performed at least three times.

### 2.4. Transmission Electronic Microscopy

Conidia were washed twice in 0.1 M cacodylate buffer and then incubated in a fixative solution (2.5% (*w*/*v*) glutaraldehyde, 2% (*w*/*v*) paraformaldehyde, 0.1 M cacodylate buffer) for 24 h at room temperature (RT) under vacuum. After washing with cacodylate buffer, samples were post-fixed for 2 h at RT in 2% osmium tetroxide. Finally, samples were dehydrated through a series of ethanol-water solutions (50%, 70%, 95% ethanol, 2 × 30 min each) and then 100% ethanol (3 × 20 min). 

For transmission electron microscopy (TEM), ethanol was replaced by propylene oxide (3 × 20 min) and samples were impregnated overnight in a propylene oxide-Epon mixture (1:1 *v*/*v*) and then in pure Epon for 16 h and 8 h. After polymerization (24 h at 37 °C, 24 h at 45 °C, and then 48 h at 60 °C), thin sections were directly examined on a JEM-1400 transmission electron microscope (Jeol, Paris, France; 120 kV). 

### 2.5. Quantification of Polysaccharide Exposure of the Conidia Cell Wall

The exposure of cell-wall polysaccharides was assessed using wheat germ agglutinin Alexa Fluor^TM^ 633 (WGA) and concanavalin A Alexa Fluor^TM^ 488 (ConA) conjugates (ThermoFisher Scientific, Illkirch-Graffenstaden, France), which bind to N-acetylglucosamine oligomers, and α-d-mannosyl and α-d-glucosyl residues, respectively. Briefly, 5 × 10^6^ conidia in HBSS were incubated with WGA or ConA at 100 µg/mL under orbital shaking, protected from light, for 30 min at 37 °C. Conidia were washed twice in HBSS and fixed with 1% PFA in HBSS before storage at 4 °C until microscopic observation. Stained conidia (20 μL) were observed under a widefield inverted fluorescence microscope (Olympus IX71) using a 20× magnification lens. Images were digitally recorded using SoftWorx software version 6.5.2. Analysis and fluorescence quantification were performed using ImageJ (Fiji) software.

### 2.6. Germination and Biofilm Assays

#### 2.6.1. Conidial Germination Assay

A standardized inoculum of 2 × 10^3^ conidia in YPD was added to each well of a 96-well microplate, which was incubated at 37 °C in 5% CO_2_ in an IncuCyte^®^ Live Cell Imaging System (Sartorius, Dourdan, France). Each well was automatically imaged every hour for 7 h. Acquisitions were then manually analyzed by counting at least 200 cells per strain at each time point. 

#### 2.6.2. Biofilm Formation

2 × 10^6^ conidia (200 µl) were transferred to YPD medium in flat-bottom 96-well polystyrene microtiter plates, centrifugated at 200 g for 2 min, and then incubated at 37 °C in 5% CO_2_ for 2 h for adhesion [36]. Supernatants were discarded to remove non-adherent conidia and 200 µL RPMI supplemented with 2% glucose and 20% fetal calf serum (FCS) was added; plates were then incubated without agitation at 37 °C in 5% CO_2_ for 24 h, 48 h, or 72 h. In parallel, blanks were prepared containing medium only. Subsequently, the supernatants were carefully discarded and the wells washed twice with PBS to remove non-adherent cells. The biofilm biomass was measured according to Mello et al. [37]. Biofilms were fixed with 200 μL 99% methanol for 15 min. After removal of the supernatant, plates were air-dried for 5 min. Then, 200 μL of 0.3% crystal violet in PBS (Sigma Aldrich) were added to each well before an incubation at RT for 20 min. The wells were washed twice with PBS to remove excess stain and the biomass then decolorized with 200 μL glacial acetic acid for 5 min. One hundred microliters of the acetic acid solution were transferred to a new 96-well plate and the absorbance measured at 590 nm using a microplate reader (SPECTROstar Nano, BMG Labtech, Champigny sur Marne, France). 

### 2.7. Resistance to Environmental Stresses

#### 2.7.1. Resistance to Ultraviolet Light

Conidia were stained with 0.1 mg/mL fluorescein isothiocyanate (FITC, Sigma-Aldrich) in carbonate buffer (0.1 M, pH = 9) at RT for 30 min, washed three times with carbonate buffer, and suspended in phosphate buffered saline (PBS). Suspensions of FITC-labelled conidia were adjusted to 5 × 10^5^/mL in uncovered sterile Petri dishes (53 mm in diameter, 6 mL) and exposed to UV-C radiation (λ = 254 nm) using a UV Stratalinker 2400 (Stratagene, La Jolla, CA, USA) at a dose of 30 mJ/cm^2^, according to the results of preliminary assays. Pellets from exposed and unexposed conidia were stained with 25 µg/mL propidium iodide (PI, Sigma-Aldrich) for 30 min at RT and protected from light. Conidia were washed with sterile water, fixed with 4% PFA, and analyzed using a LSRFortessa^TM^ cytometer (BD Biosciences, Le-Pont-de-Claix, France). Each replicate included a negative control, reflecting live conidia (unexposed FITC^+^ conidia), and a positive control consisting of heat-killed conidia (85 °C, 20 min). Both controls were used to set the gates for the positivity of each marker. Live conidia were FITC^+^ PI^−^ and the dead conidia were double-positive (FITC^+^ PI^+^). The percentage of live conidia was determined as the percentage of PI^−^ conidia among FITC^+^ conidia. The reduction in conidia survival was calculated relative to control conidia not exposed to UV (viability control) as: 100*((% of live conidia among unexposed conidia—% of live conidia among exposed conidia)/% of live conidia among unexposed conidia). 

#### 2.7.2. Resistance to Heat Shock

Conidial suspensions adjusted to 10^3^ cells/mL were incubated at 40 °C or 50 °C for 15 min. After homogenization, 200 µL conidia suspension were inoculated in triplicate onto PDA plates. The survival rate was calculated by counting the number of colonies after incubation at 37 °C for 72 h and defined as the ratio of the colony number between exposed and control (unexposed) conditions.

#### 2.7.3. Growth under Oxidative Conditions

The ability to grow in the presence of oxidizing compounds, i.e., menadione (Sigma Aldrich, stock solution prepared in DMSO) or cumene hydroperoxide (CHP, Sigma Aldrich), was studied by nephelometry [38]. To study hyphal growth in liquid media, 10^5^ conidia were inoculated into microplate wells containing 40 µM menadione or 200 µM cumene hydroperoxide in potato dextrose broth (PDB). Fungal growth was monitored automatically for 40 h at 37 °C using a laser-based microplate nephelometer (NEPHELOstar, BMG Labtech). For each condition, the area under the growth curve representative of the lag phase was used to calculate the percentage of growth under the oxidative condition relative to the standard growth condition (PDB medium). At least three replicates were conducted per treatment. 

### 2.8. Cell Wall Integrity Assay

The growth phenotypes of the parental strains (WT and Δ*KU70)* and their disruptants were assessed on PDA supplemented with 1 mg/mL Congo red or 0.0064% sodium dodecyl sulfate (SDS, purchased from Sigma Aldrich). Concentrations were chosen according to the results of preliminary assays. A growth control on PDA was added in each experiment. Plates were incubated at 30 °C for seven days. The colony diameter, reflecting the growth rate, was expressed relative to the growth on control PDA as follows: (100–100*(diameter on PDA − diameter on PDA + stressor)/diameter on PDA]). Each test was repeated three times, with four replicates each time.

### 2.9. Antifungal Susceptibility Testing

In vitro susceptibility testing was performed using broth microdilution for filamentous fungi according to EUCAST guidelines with concentrations ranging from 0.016 µg/mL to 8 µg/mL. The antifungals used were amphotericin B (AmB), voriconazole (VRC), itraconazole (ITC), posaconazole (POS), and anidulafungin (ANI), all purchased from Sigma-Aldrich, reconstituted in DMSO and stored frozen at −80 °C until use. The MICs were determined in flat-bottomed 96-well plates with conidial suspensions prepared in RPMI 1640 supplemented with 2% glucose, buffered with MOPS, and adjusted to a final concentration of 5 × 10^5^ CFU/mL, as previously described [39]. Inoculated plates were incubated for 72 h at 35 °C. MICs were spectroscopically determined at 405 nm using a spectrophotometer (NEPHELOstar, BMG Labtech) as the antifungal concentration that resulted in 90% (AmB) or 50% growth inhibition (VRC, ITC, POS, ISA).

### 2.10. Transcriptome Analysis

For transcriptome sequencing, 10^8^ *S. apiospermum* conidia from the Δ*KU70* strain and its M1 mutant were incubated in YPD medium under continuous agitation (300 rpm) at 37 °C for 30 h. Mycelia were harvested, immediately frozen in liquid nitrogen, and stored at −80° until RNA extraction. Four biological replicates were performed. Fifty milligrams of mycelium sprayed with liquid nitrogen were then subjected to total RNA extraction using the Plant/Fungi Total RNA Purification kit (Norgen Biotek, Thorold, Canada), with additional DNase treatment with TURBO DNase (Thermo Fisher Scientific) to remove residual genomic DNA. Total RNA was checked for integrity using a Bioanalyzer system (Agilent) and quantified using the fluorescent-based quantification Qubit dsDNA HS Assay Kit (Thermo Fisher Scientific).

Directional libraries were prepared from rRNA-depleted RNA using the TruSeq Stranded mRNA sample Preparation Kit following the manufacturer’s instructions (Illumina, Evry-Courcouronnes, France). Libraries were checked for quality on Bioanalyser DNA chips (Agilent, Les Ulis, France) and quantified using the fluorescent-based quantification Qubit dsDNA HS Assay Kit. RNA sequencing was performed on the Illumina NextSeq 500 platform using a High Output Kit (75 cycles). 

RNA-seq analysis was performed using Sequana 0.12.7 [40]. In particular, we used the RNA-seq pipeline 0.15.0 (https://github.com/sequana/sequana_rnaseq) built on top of Snakemake 6.7.0 [41]. Reads were trimmed from adapters using fastp 0.20.1 [42] and mapped to the *S. apiospermum* reference genome (GCF_000732125.1) using STAR 2.7.8a [43]. FeatureCounts 2.0.1 [44] was used to produce the count matrix and assign reads to features using annotation from NCBI GCF_000732125.1 with strand-specificity information. Pseudogenes were considered to be genes. Quality control statistics were performed using MultiQC 1.11 [45]. Statistical analysis of the count matrix was performed to identify differentially regulated genes, comparing M1 to Δ*KU70*. Transcriptomic profiles were clustered using principal component analysis (PCA). Differential expression was determined using DESeq2 library 1.30.0 [46], indicating the significance (Benjamini-Hochberg adjusted *p*-values, false discovery rate FDR < 0.05) and effect size (fold-change).

Gene annotation was performed to analyze the functions of the DEGs using Blast2GO for gene ontology (GO) annotation and GO for functional classification. 

Additionally, Interproscan (https://www.ebi.ac.uk/, accessed on 1 April 2022) was used for the functional analysis of proteins, classifying them into families and predicting domains based on several databases, including the Pfam, CDD, and Panther databases.

For quantitative real-time PCR analysis, RNA was reverse transcribed to cDNA using a high-capacity cDNA reverse transcription kit (Applied Biosystems, Illkirch-Graffenstaden, France) according to the manufacturer’s instructions. Quantitative PCR amplifications were carried out in duplicate using PowerUp SYBR^®^ green PCR master mix (Applied Biosystems), 0.3 μM primers, and 2 μL of cDNA in a final volume of 10 μL, in 384-well optical plates, using a CFX384 OPUS^TM^ real-time PCR detection system (Bio-Rad, Marnes-la-Coquette, France). Gene-specific primers (Table S1) were synthesized by Eurogentec. Expression levels of target genes were normalized by comparison to the expression of the *S. apiospermum UBC6* and *FIS1* housekeeping genes. Results are expressed as 2^−ΔΔCq^, referring to the fold induction in the T, M1, and M2 strains relative to the mean quantification cycle obtained with the parental strains.

### 2.11. In Vitro Macrophage Infection Assays

#### 2.11.1. Macrophage Preparation

Human peripheral blood mononuclear cells (PBMCs) were isolated from blood buffy coats obtained from healthy donors supplied by the Etablissement Français du Sang, Rennes, France. PBMCs were isolated by density gradient centrifugation and CD14^+^ cells (monocytes) were isolated by positive magnetic cell-sorting (Miltenyi Biotec). Monocytes (2 × 10^6^) were plated in 12-well Nunc UpCell plates (ThermoFisher Scientific) and differentiated into macrophages in complete medium (RPMI 1640, Gibco, Life Technologies, Villebon sur Yvette, France), 10% decomplemented (FCS), 100 IU/mL penicillin, and 100 µg/mL streptomycin) supplemented with 10 ng/mL granulocyte-macrophage-colony stimulating factor (GM-CSF) (Miltenyi, Biotec, Paris, France) for five days, at 37 °C in 5% CO_2_. 

#### 2.11.2. Phagocytosis and Killing Assays

After differentiation, macrophages were plated at 5 × 10^5^ cells per well in 24-well plates and infected with FITC-labelled conidia with a MOI of 5:1 for the ingestion assay and 1:1 for killing experiments. Plates were centrifugated at 500 g for 5 min at RT to synchronize phagocytosis and incubated at 37 °C in 5% CO_2_, protected from light, for 2 h (phagocytosis) or 6 h (phagocytosis and killing). Plates were then put on ice to block macrophage activity, the supernatants removed, and the cells washed three times with cold DPBS to remove extracellular non-adherent conidia.

- Phagocytosis assay:

Cells were removed from the wells by gentle mixing and transferred to Eppendorf tubes, washed once with DPBS, and centrifuged at 500 g for 5 min. Macrophages were then transferred into 96-well U-bottom plates for labelling. Cells were stained for viability using the LIVE/DEAD™ Fixable Yellow Dead Cell Stain Kit (ThermoFisher Scientific) for 30 min at RT, protected from light. After washing, Fc receptors were blocked with Fc-R Blocking Reagent (Miltenyi) for 10 min at 4 °C and marked with anti-CD11b/Mac1 (Mouse anti-human, BD horizon) for 15 min at 4 °C, washed twice with PBS-2% SVF, fixed with 4% PFA, and analyzed on a LSRFortessa^TM^ cytometer (BD Biosciences) to quantify the percentage of macrophages with ingested conidia. Data were analyzed using Flowlogic software according to the gating process previously designed to remove dead macrophages and distinguish between the CD11b^+^/FITC^−^ population (non-infected macrophages) and the double-positive CD11b^+^/FITC^+^ population (macrophages with adherent and/or ingested conidia) [47].

- Killing assay

After 6 h of incubation, cells were immediately lysed using cold sterile water and incubated at 4 °C for at least 1 h or overnight. The well contents were then transferred into Eppendorf tubes and centrifuged at 10,000× *g* for 5 min. Pellets were then stained with PI and analyzed as described above (see Resistance to Ultraviolet Light).

#### 2.11.3. Cytokine Assays

Primary macrophages were plated in 24-well plates at 10^6^ cells/mL and incubated with 10^7^ (MOI 10:1) live conidia in complete RPMI for 12 h at 37 °C in 5% CO_2_. Supernatants were then collected and immediately stored at −80 °C. Pro-inflammatory cytokines were measured using a 13 plex-bead-based immunoassay LEGENDplex^®^ multi-analyte flow assay (BioLegend, Paris, France) according to the manufacturer’s instructions. Cytokine concentrations were determined using standard curves obtained using recombinant cytokine standards provided in the kit. Samples were analyzed on a LSRFortessa^TM^ cytometer (BD Biosciences) and the data processed using the online platform provided by the kit manufacturer. 

### 2.12. Statistical Analysis

All experiments were performed in triplicate or quadruplicate at least three times independently. Data are expressed as the mean ± standard error of the mean (SEM). Statistical analysis was performed using GraphPad Prism software (GraphPad Software, Inc., La Jolla, CA, USA). A one-way analysis of variance Kruskall-Wallis test or a Mann-Whitney test were used to evaluate differences between strains. In all analyses, *p*-values ≤ 0.05 were considered statistically significant. 

## 3. Results

### 3.1. Disruption of the PIG1 Gene in S. apiospermum

**Figure 2:**
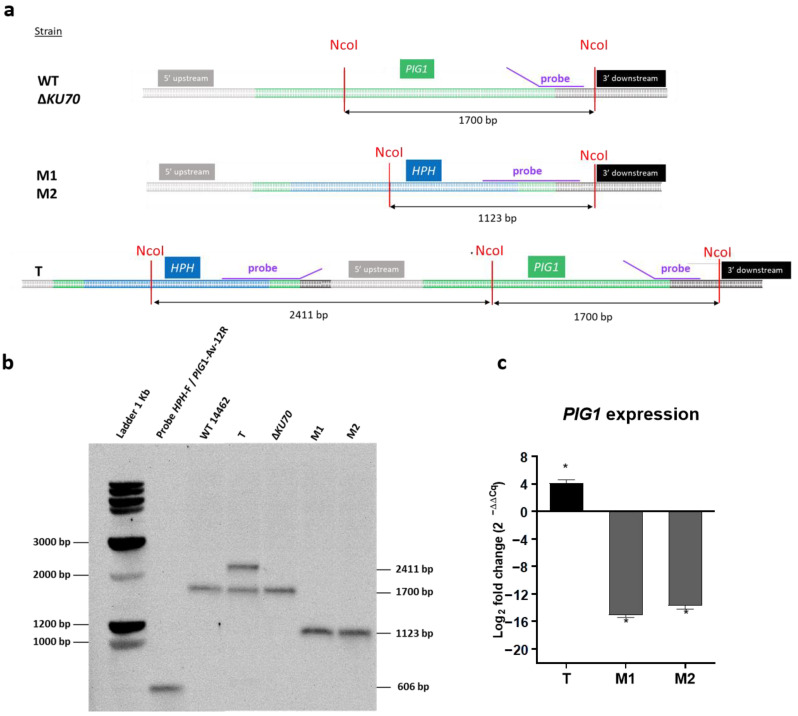
Generation of *S. apiospermum* Δ*PIG1* mutants. (**a**) Restriction map of the *S. apiospermum PIG1* locus and strategy for the construction of the disruption cassette. *Nco*I restriction sites are identified and the purple line indicates the hybridization site of the probe. The size of the expected fragments is indicated by the arrows. (**b**) Southern-blot analysis of *PIG1* transformant strains. Genomic DNA of all strains (parental and mutant derivatives) were digested by *Nco*I and probed with a hybridization probe corresponding to a 600-bp fragment including the *PIG1* 3′ flanking region and 3′ end of the *HPH* gene. The expected signals were 1700 bp for the parental strains and 1123 bp for the M1 and M2 mutants. Two unexpected bands were observed for the T strain (2411 bp and 1700 bp), suggesting integration of the repair cassette upstream of the *PIG1* gene. (**c**) Expression of *PIG1* mRNA by the three HPH-resistant transformants relative to their respective parental strain (i.e., T vs. WT, M1 vs. Δ*KU70*, and M2 vs. Δ*KU70)*. After reverse transcription, mRNA expression was quantified by quantitative PCR. Data were normalized to the expression of the *UBC6* and *FIS1* housekeeping genes and are representative of four independent experiments. Significant differences are indicated by asterisks: * *p* ≤ 0.05 (Mann-Whitney test). T: transformant derived from the WT strain; M1 and M2: transformants derived from the Δ*KU70* strain.

The CRISPR/Cas9 technology recently adapted to *S. apiospermum* was used to disrupt the *PIG1* gene. Because of the low frequency of the homologous recombination, we performed the experiment in the wild-type strain (WT 14462), as well as in the previously developed non-homologous end joining-deficient strain Δ*KU70* [33]. Three hygromycin-resistant strains corresponding to three putative *PIG1* disruptants were obtained, one derived from the WT strain (T strain) and two derived from the Δ*KU70* strain (Δ*KU70*/Δ*PIG1*-1 (M1) and Δ*KU70*/Δ*PIG1*-2 (M2)). The genotype of the monosporic isolates was verified by Southern blotting. A band of expected size (1123 bp) was visualized for M1 and M2 after probe hybridization, instead of the 1700-bp band observed for the parental strain (Figure 2a,b). Replacement of the *PIG1* gene by the repair cassette was confirmed by DNA sequencing (data not shown) and *PIG1* mRNA expression was completely repressed (Figure 2c). By contrast, two bands (2411 bp and 1700 bp) were observed in the T strain. This result, associated with DNA sequencing, showed integration of the repair cassette 782 bp upstream of the *PIG1* gene. No mutation was found in the *PIG1* coding sequence, which could have possibly resulted in a reading-frame shift. Moreover, *PIG1* mRNA was overexpressed in the T strain relative to the parental WT strain (Figure 2c). 

### 3.2. Melanin Content and Cell-Wall Structure

**Figure 3:**
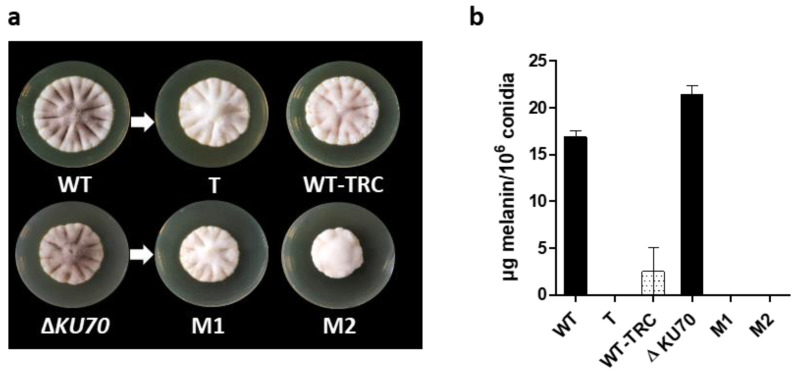
Detection of melanin in parental and mutant *S. apiospermum* strains. (**a**) Macroscopic aspect of parental and mutant strains after nine days at 37 °C on PDA medium. WT-TRC indicates the WT strain grown on a PDA plate supplemented with 50 µg/mL tricyclazole as a specific inhibitor of melanin biosynthesis. (**b**) Melanin quantity per million conidia, extrapolated from a standard curve of synthetic melanin, determined by EPR (Graph representative of at least three independent measurements). T: transformant derived from the WT strain; M1 and M2: transformants derived from the Δ*KU70* strain.

In contrast to the dark brown pigment observed in the parental strains (Figure 3a), colonies from the three transformants showed a similar non-melanized white phenotype. The color defect did not result from an inability to sporulate, as supported by the presence of conidia by microscopic observation. Moreover, based on the paramagnetic structure of DHN-melanin, the quantification of conidia melanin could be assessed by electronic paramagnetic resonance (EPR), and confirmed the absence of a melanin-related EPR signal in conidia from the T, M1, and M2 strains (Figure 3b). These results indirectly show that the transcription factor PIG1 regulates the expression of (a) gene(s) of the DHN-melanin cluster. Thus, the *PIG1* mRNA overexpression observed in the T strain did not correlate with a functional transcription factor. This mutant was included in the subsequent experiments to decipher the role of DHN-melanin in *S. apiospermum* as the Southern blot confirmed that there was no ectopic integration elsewhere in the genome and as EPR confirmed the absence of DHN-melanin in the T strain.

Of note, tricyclazole (TRC) was previously shown to be a fungicide that specifically inhibits both 1,3,6-trihydroxynaphtalene and 1,3,6,8-tetrahydroxynaphtalene reductases at nontoxic concentrations [48,49]. TRC is thus commonly used as a melanin biosynthesis inhibitor in studies focusing on DHN-melanin function. Here, the light reddish-brown pigment of the WT strain grown in the presence of TRC, similar to previous descriptions, reflects the accumulation of melanin shunt intermediates, confirming the inhibitory effect of TRC on DHN-melanin biosynthesis in *S. apiospermum* (Figure 3a).

**Figure 4:**
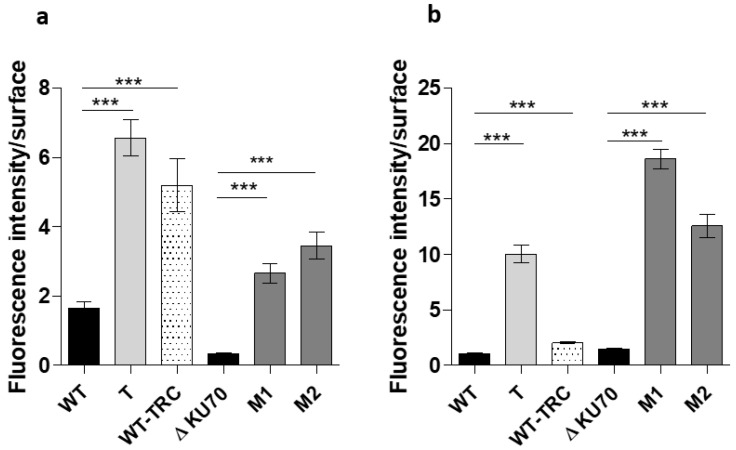
Analysis of the fluorescence labelling of conidia surface carbohydrates with FITC-conjugated lectins. (**a**,**b**) Fluorescence intensity of (**a**) ConA binding to mannosyl and glucosyl residues and (**b**) WGA binding to N-acetylglucosamine (NAG) residues on the surface of the conidia. Fluorescence analysis was performed using ImageJ (Fiji) software from at least 200 conidia. The graph is representative of three independent experiments. Significant differences are indicated: *** *p* < 0.001 (Kruskall-Wallis test). T: transformant derived from the WT strain; M1 and M2: transformants derived from the Δ*KU70* strain.

In the absence of the external melanin layer in the T, M1, and M2 conidia, the cell wall content was expected to be different, or at least disorganized. We thus investigated the accessibility of the cell wall carbohydrates to mannose-binding and N-acetylglucosamine-binding lectins (ConA and WGA, respectively). Most of the parental cells were not labeled, whereas almost all conidia from the mutant strains were intensely labeled, together with the WT-TRC conidia, suggesting polysaccharide unmasking by the removal of the melanized outer layer (Figure 4 and Figure S1). 

**Figure 5:**
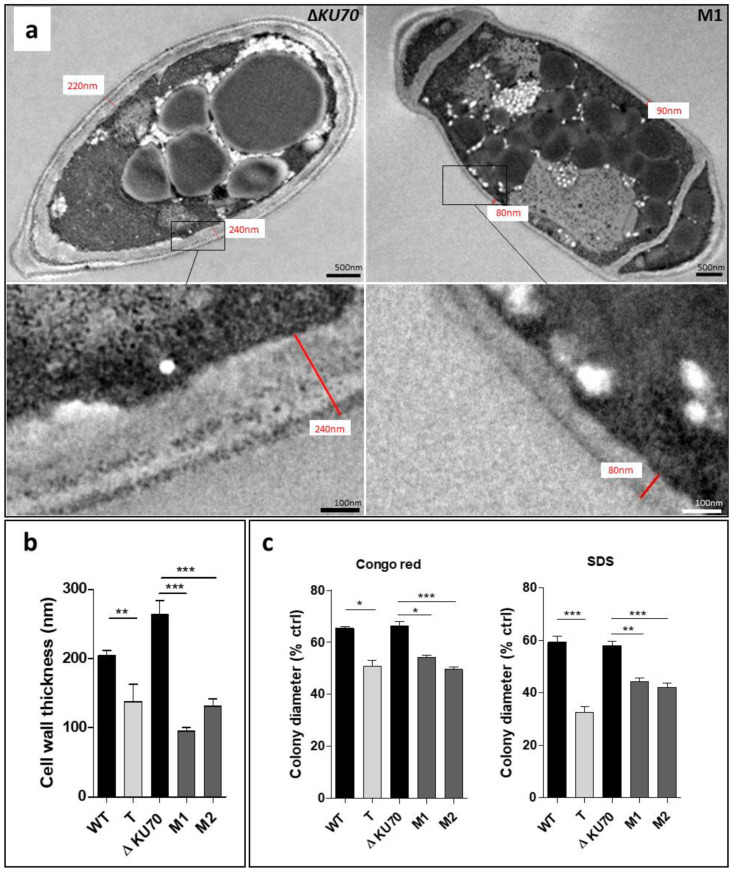
Evaluation of cell-wall thickness and integrity. (**a**) TEM of Δ*KU70* and M1 conidia cell walls. The image is representative of the three transformants. (**b**) Thickness determined from transmission electron microscopy (TEM) acquisition for 20 acquisitions. (**c**) Growth in contact with the cell-wall chemical stressors Congo Red and SDS. After an incubation of seven days at 30 °C, the colony diameter was measured and expressed according to the control growth on stress-free PDA. All graphs include results obtained from three independent experiments. Significant differences are indicated: * *p* < 0.05, ** *p* < 0.01, *** *p* < 0.001 (Kruskall-Wallis test). T: transformant derived from the WT strain; M1 and M2: transformants derived from the Δ*KU70* strain.

In addition, TEM examination showed the mean cell wall thickness to be significantly less for the three disruptants (T, M1, and M2) than that of the parental strains by a factor of 1.5 (*p* < 0.01), 2.7 (*p* < 0.001), and 2.0 (*p* < 0.001), respectively (Figure 5a,b). We examined the cell wall integrity of transformants by assessing the effect of cell-wall perturbing agents through the measurement of mycelial growth on PDA plates [50]. Growth of the T, M1, and M2 strains in contact with Congo red and SDS was significantly less than that of the WT and Δ*KU70* strains (Figure 5c), supporting the role of PIG1 in cell-wall structure and integrity.

### 3.3. Cell Morphology, Germination and Biofilm Formation

**Figure 6:**
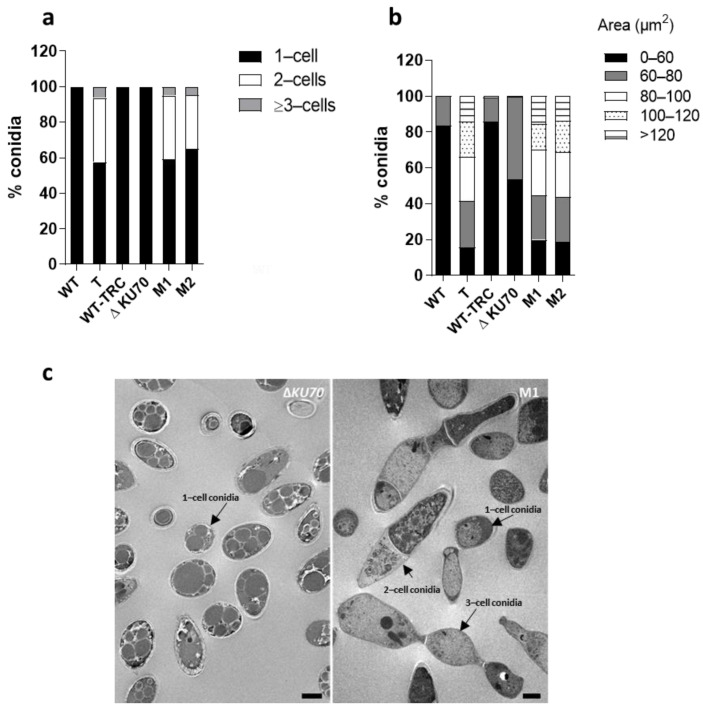
Morphology of conidia. (**a**) Distribution of conidia morphotypes. At least 200 conidia were measured. (**b**) Distribution of conidia sizes according to cross-sectional area. At least 200 conidia were measured from images of conidia suspensions acquired with a widefield inverted fluorescence microscope (Olympus IX71) at 20× magnification and analyzed using ImageJ (Fiji) software. (**c**) Transmission electron microscopy (TEM) of Δ*KU70* and M1 conidia. Images are representative of the three transformants. Scale bar: 2 µm. T: transformant derived from the WT strain; M1 and M2: transformants derived from the Δ*KU70* strain.

In addition to the loss of the external melanin coating, conidia from the T and M1 and M2 recombinants showed significant changes in terms of shape and structure. Unexpectedly, the recombinant strains produced multicellular cross-sectioned spores with one or two septa, accounting for 35 to 43% of the total conidia (Figure 6a,c). The remaining spores from recombinants were similar in shape, although a portion appeared to be more elongated than those of the parental strain, corresponding to the fraction of 1-cell conidia displaying a size comprised between 80 and 100µm (Figure 6b). By contrast, the shape of WT-TRC conidia remained unchanged (Figure 6a). The results suggest that the transcription factor PIG1 not only regulates genes of the DHN-melanin cluster but may also indirectly regulate the expression of other genes. 

**Figure 7:**
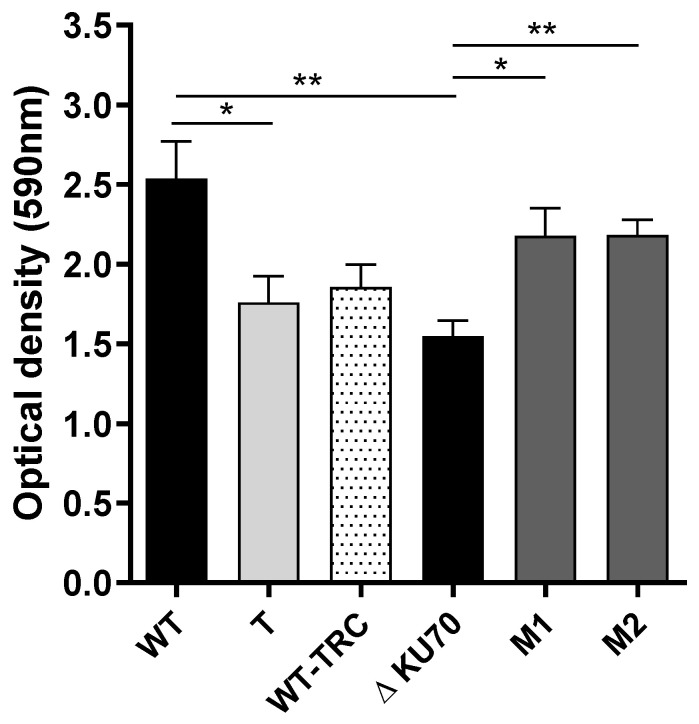
Biofilm formation. The total biomass was monitored using a crystal violet assay. Results from three independent experiments are shown. Significant differences are indicated as follows: * *p* < 0.05, ** *p* < 0.01 (Kruskall-Wallis test). T: transformant derived from the WT strain; M1 and M2: transformants derived from the Δ*KU70* strain.

We also investigated germination and biofilm formation. Although the kinetics of germ-tube formation were slightly slower in the T strain, it was not hampered in the two Δ*KU70*-derived transformants relative to their parental strain (data not shown). Similarly, the ability of both M1 and M2 to adhere and grow on inert material appeared to be significantly enhanced with respect to the Δ*KU70* strain (+40%, *p* < 0.05), as shown by the amount of biomass after 24 h of incubation on a polystyrene plate (Figure 7). By contrast, the growth of the T strain appeared to be less than that of the WT strain. The metabolic activity within the biofilms remained unchanged (data not shown). Of note, however, the mutant strains exhibited a similar ability to form biofilms and the differences observed upon comparing each mutant to its parental strain appeared to be mainly based on different characteristics of the two parental strains.

### 3.4. Resistance to Exogenous Stress

**Figure 8:**
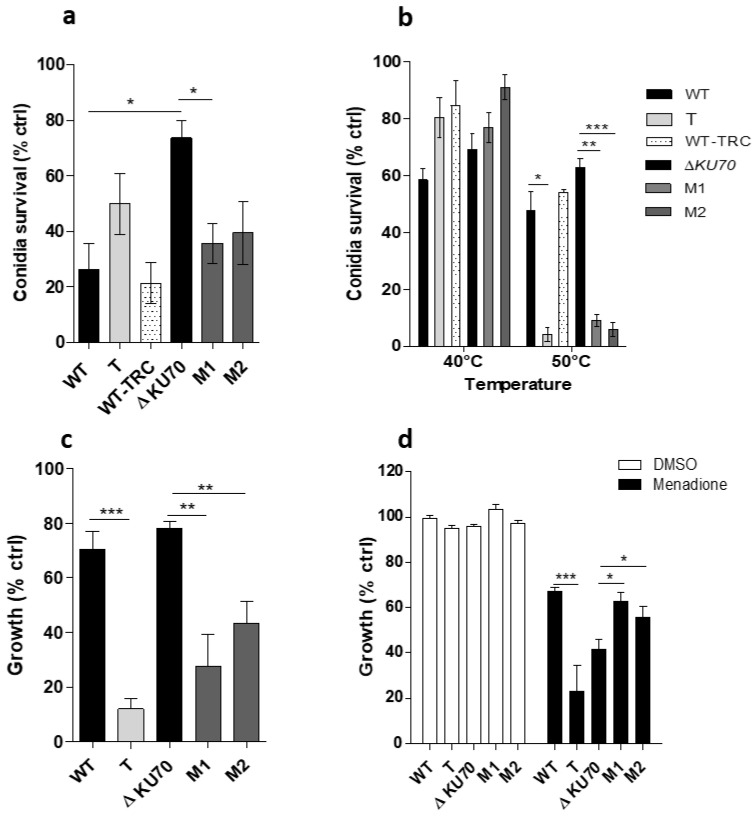
Susceptibility of the T and the M1 and M2 deleted strains exposed to exogenous stresses. (**a**) Conidia survival after exposure to UV-C light. After exposure to a dose of 30 mJ/cm^2^ at 254 nm, conidia were stained with 25 µM propidium iodide (PI) before being analyzed on a LSRFortessa^TM^ cytometer. The graph presents the percentage survival among exposed FITC^+^ conidia expressed relative to that of unexposed FITC^+^ conidia (live control), as follows: 100× ((% PI^−^ FITC^+^ conidia among unexposed control conidia—% PI^−^FITC^+^ conidia among exposed conidia)/(% PI^−^FITC^+^ conidia among unexposed control conidia)). (**b**) Conidia survival after exposure to high temperature. After incubation at 40 °C or 50 °C for 15 min, 200 conidia were incubated on PDA plates at 37 °C for 72 h. The number of colonies reflect the number of live conidia. The graph presents the ratio between the number of colonies recovered after exposure of the conidia to heat and the number of colonies recovered after no heat exposure on PDA plates. (**c**,**d**) Growth under oxidative conditions. Mycelial growth in PDB medium supplemented with 200 µM cumene hydroperoxide (**c**) or 40 µM menadione (**d**) was monitored when incubated for 40 h at 37 °C by nephelometry. Results are expressed in relative nephelometric units resulting from the measurement of the intensity of scattered light through the wall as an indication of the increasing turbidity due to the mycelia. DMSO was used to dissolve the menadione powder. Graphs present the relative growth calculated as the ratio between the nephelometric signal obtained in the presence of an oxidizing compound and in oxidant-free PDB (growth control). All graphs include results obtained from three independent experiments: * *p* < 0.05, ** *p* < 0.01, *** *p* < 0.001 (Kruskall-Wallis test). T: transformant derived from the WT strain; M1 and M2: transformants derived from the Δ*KU70* strain.

Many studies have shown that melanized fungi are more resistant to stressful conditions than non-melanized species, enabling them to survive under extreme environmental conditions. Thus, we exposed the mutant and parental strains to deleterious conditions to determine whether unpigmented mutant conidia were more vulnerable to environmental stress. First, exposure of the strains to UV-C light showed the survival rate of M1 and M2 conidia to be only 35.6% and 39.4% that of unexposed conidia, respectively, whereas survival of the parental Δ*KU70* strain was 73.6% relative to the unexposed controls (Figure 8a). By contrast, there was no significant difference in the survival of the WT-derived T strain nor the TRC-treated WT strain relative to that of the parental WT strain. This may be related to the difference in susceptibility of the parental strains, as suggested by the three-fold lower viability of the WT strain relative to that of Δ*KU70.*

Moreover, although incubation at 40 °C for 15 min had no significant effect on conidia viability, the survival rate of the three mutants markedly dropped after exposure to 50 °C to 4.1% (*p* < 0.05), 9.1% (*p* < 0.01), and 5.9% (*p* < 0.001), respectively, whereas 47.5% to 62.8% of the parental strain conidia survived (Figure 8b). The survival of WT-TRC conidia was similar to the WT strain. Similarly, we also evaluated the ability of the T, M1, and M2 strains to grow in oxidizing medium by nephelometry. The three mutant strains showed a large reduction in growth in cumene hydroperoxide relative to growth in PDB (control medium): a reduction of 88.0% for the T (*p* < 0.001), 72.5% for the M1 (*p* < 0.01), and 56.8% for the T (*p* < 0.01) strains. By contrast, the parental strains were able to maintain more than 70% of their growth in the reference medium (Figure 8c). When exposed to menadione, the relative growth of the T strain was lower than that of the WT strain, which contrasted with the growth of both the M1 and M2 strains, which was slightly enhanced relative to that of the Δ*KU70* strain (*p* < 0.05) (Figure 8d). 

### 3.5. Antifungal Susceptibility

The fungal cell wall, composed of molecules not present in humans, constitute an ideal target for the development of clinical antifungal compounds. Therefore, modifications of the cell wall may influence responses to antifungal drugs. The susceptibility of mutants to various antifungals was assessed by the broth microdilution method according to EUCAST recommendations (Table 1). Amphotericin B had a deleterious impact on the growth of both the M1 and M2 recombinants, shown by the lower mean MICs of 0.58 ± 0.38 and 0.83 ± 0.29 µg/mL, respectively, relative to that of for Δ*KU70*, which was resistant to AmB (MIC > 8 µg/mL). By contrast, both the T and WT strains showed similar MICs of approximately 0.5 µg/mL. These results were further supported by E-test susceptibility testing (not shown). The susceptibility to triazole compounds and anidulafungin were unchanged between the parental strains and their transformants (Table 1). 

### 3.6. Transcriptomic Analysis of M1 Transformant

We next conducted a comparative transcriptomic analysis between the MI strain and its parental Δ*KU70* strain to understand the involvement of the transcription factor *PIG1* in melanin biosynthesis and the phenotypic characteristics described above, especially relative to the morphogenesis of the *S. apiospermum* cell wall. Mycelia were harvested from YPDA medium after 30 h of incubation and RNA extracted and purified for transcriptome sequencing. In total, eight biological samples (four biological replicates) were thus sequenced by next-generation sequencing. After a stringent quality check and data cleaning, 9.4 to 15 million assigned reads were obtained for each sample (Table S2), which were further spliced into 10,919 assembled unigenes. 

In addition to biostatistical quality controls, RNA-seq based differential gene expression data were further validated by RT-qPCR for eight selected representative genes, resulting in an excellent agreement between the fold change values determined by global sequencing and specific qPCR (Table 2).

**Figure 9:**
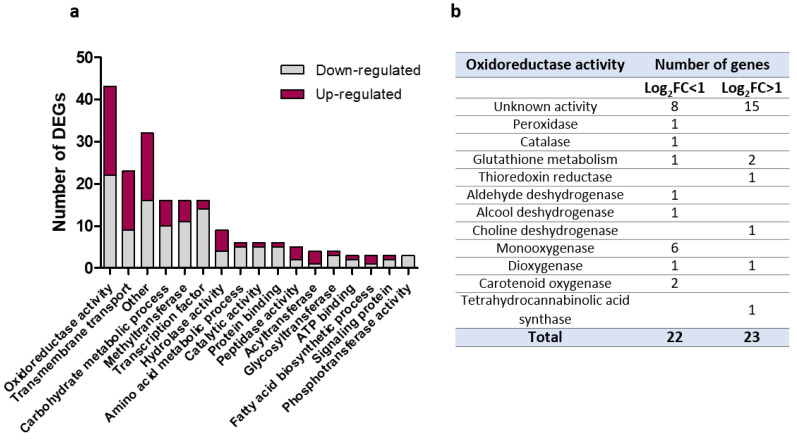
Functions of differentially expressed genes (DEGs) in M1 relative to Δ*KU70*. (**a**) Distribution of functions or metabolic pathways in DEGs with a known function (N = 198). The 80 DEGs with unknown function are not included. (**b**) Detailed functions of DEGs involved in an oxidoreduction process.

In total, 416 differentially expressed genes (DEGs) were obtained for M1 relative to Δ*KU70,* regardless of the fold change value (FC). Considering only DEGs with a |log_2_FC| > 1 (adjusted *p*-value < 0.05) resulted in 278 DEGs, including 78 pseudogenes, of which 115 were upregulated and 163 downregulated. Only 110 genes (39.6%) were assigned with GO terms, including biological processes (n = 67), molecular functions (n = 107), and cellular components (n = 41). In addition, we performed a functional analysis of the resulting proteins using Interproscan, making it possible to associate a function or metabolic pathway with 198 DEGs (71.2%), of which 83 were upregulated and 115 downregulated (Figure 9a). 

Among DEGs with a known function found in M1, the five most represented functions were associated with oxidoreduction processes (N = 43), transmembrane transport (N = 23), carbohydrate metabolic processes (N = 16), transcription factors (N = 16), and methyltransferases (N = 16) (Figure 9a). The significant representation of oxidoreduction enzymes among DEGs in the M1 transformants could at least partially explain some of the phenotypic observations with the recombinant. For example, a number of key proteins involved in conidial protection against oxidative injuries, such as peroxidases, catalases, or glutathione enzymes, appeared to be differentially expressed (Figure 9b). Dysregulated enzymes were also involved in cell-wall metabolism included, for example, various glycosidases, a polysaccharide synthase (log_2_FC = −1.2, *p* = 9.6 × 10^−5^) and a neutral/alkaline non-lysosomal ceramidase (log_2_FC =- 1.45, *p* = 0.038). Other DEGs potentially related to conidiogenesis were also identified in M1, such as the genes encoding the membrane protein SUR7/RIM9-like (log_2_FC = 2.09, *p* = 0.0024) and conidiation-specific protein-6 (log_2_FC = −3.18, *p* = 0.030) (data not shown).

**Figure 10:**
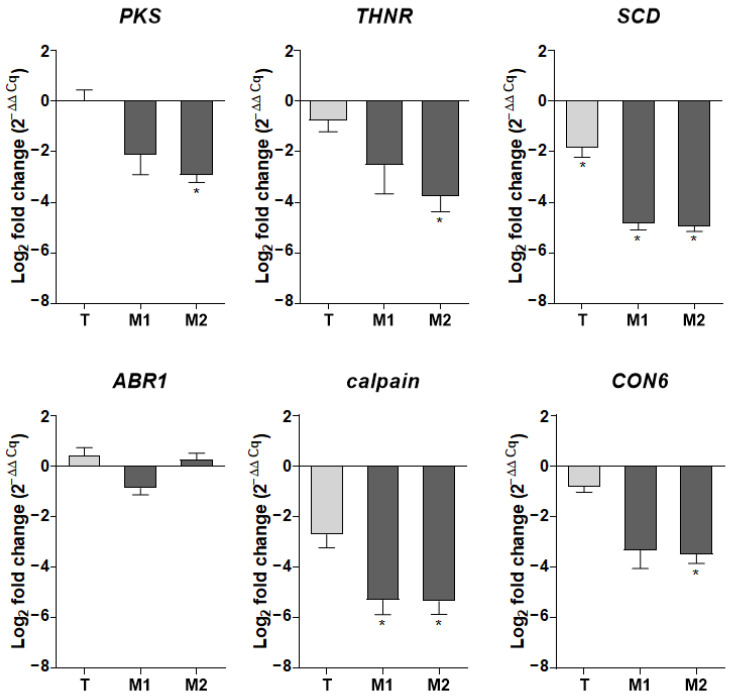
Fold-induction mRNA values for a selected panel of genes in the T, M1, and M2 strains relative to their parental Δ*KU70* strain. Expression levels of target genes were normalized against the expression of the *S. apiospermum UBC6* and *FIS1* housekeeping genes. Results are expressed as 2^−ΔΔCq^, referring to the fold induction in the T, M1, and M2 strains relative to the mean quantification cycle obtained with the parental strains. Data are from four biological replicates: * *p* ≤ 0.05 (Mann-Whitney test). T: transformant derived from the WT strain; M1 and M2: transformants derived from the Δ*KU70* strain.

The expression of genes selected for the qPCRs performed to validate the M1 RNA-seq data was also explored in the M2 and T strains (Figure 10). Interestingly, the scytalone dehydratase-encoding gene was the only melanin biosynthesis-related gene found to be downregulated in all three transformants. The repression of this key-enzyme from the melanin biosynthesis cascade supports the melanin-deficient phenotype observed for the three recombinants. 

### 3.7. In Vitro Macrophage Infection Assays

**Figure 11:**
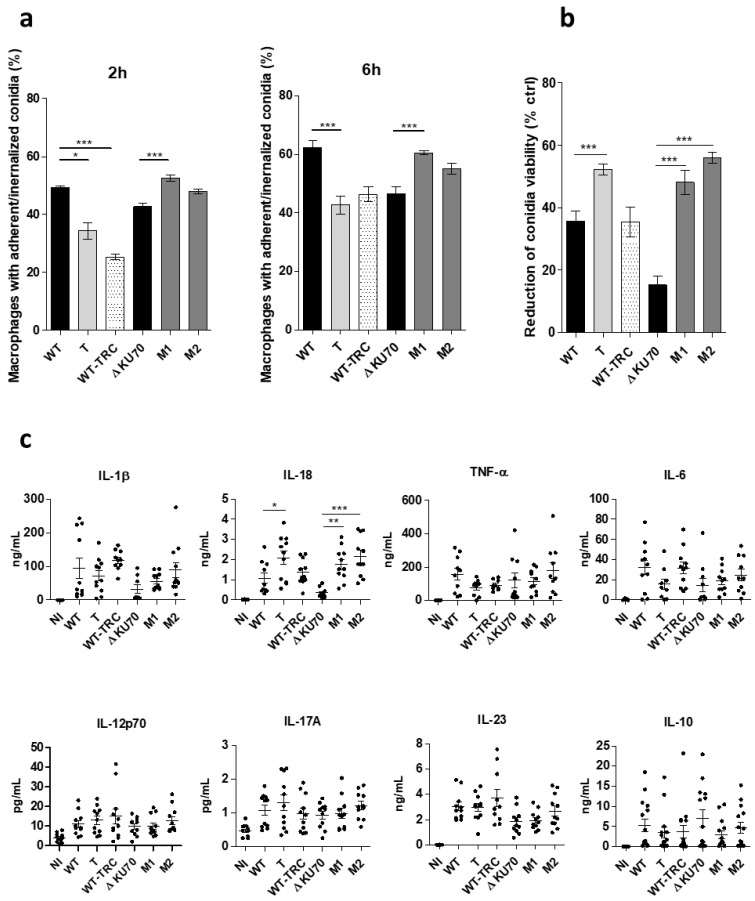
Phagocytosis, killing, and cytokine release from human macrophages infected with T, M1, and M2 conidia. (**a**) Percentage of macrophages with adherent or ingested conidia after 2 or 6 h of incubation with FITC-stained conidia (MOI 5:1) at 37 °C in 5% CO_2_. Graphs present the percentage of FITC^+^ CD11b^+^ events among CD11b^+^ cells (macrophages). (**b**) Conidia killing after ingestion by macrophages. After 6 h of incubation (MOI 1:1), extracellular conidia were removed by three PBS washes and internalized conidia were released by macrophage lysis (cold water). The graph presents the decrease in conidia viability among ingested conidia relative to the viability of conidia not exposed to macrophages (live control). Conidia viability was determined as the percentage of FITC^+^ PI^–^ events among FITC^+^ events (total conidia). The reduction in conidia survival was calculated relative to control conidia as: 100 × ((% of live conidia among control conidia − % of live conidia among cell-exposed conidia)/% of live conidia among control conidia). (**c**) Cytokine release in culture supernatants from macrophages incubated with conidia for 12 h (MOI 10:1). Concentrations were determined using a 13 plex-bead-based LEGENDplex^®^ multianalyte flow assay. A non-infected (NI) condition was included in each experiment. Data are from three independent experiments. * *p* < 0.05, ** *p* < 0.01, *** *p* < 0.001 (Kruskall-Wallis test) T: transformant derived from the WT strain; M1 and M2: transformants derived from the Δ*KU70* strain.

The first step of the host immune response to fungal conidia has been shown to be highly dependent on the early recognition of cell-wall specific patterns by phagocytic cells. Thus, the objective of these experiments was to assess the response of human macrophages exposed to conidia from the three melanin-deficient transformants. In particular, a phagocytosis assay was conducted to determine whether cell-wall sugar unmasking was associated with easier recognition, binding, and subsequent conidia internalization by macrophages. Both M1 and M2 recombinants showed a higher ability to adhere to and be internalized by macrophages, as at 6 h post-infection, the percentage of FITC^+^ macrophages infected with M1 and M2 reached 60.5% (*p* < 0.001) and 55.1% (ns), respectively, vs. only 46.5% of Δ*KU70* infected cells (Figure 11a). Different results were obtained with the WT-derived strains, as the percentages of infected macrophages were significantly lower at 2 and 6 h when challenged with the T transformant and the TRC-treated WT strain than with the WT strain: 34.3% (*p* < 0.05) and 25.4% (*p* < 0.001) vs. 49.3% 2 h post-infection. Although the phagocytosis assay resulted in contrasting conclusions between the WT and Δ*KU70*-derived strains, the ability of recombinant conidia to survive macrophage killing was equally impeded, as shown by the decrease in conidia viability of approximately 50% for the three transformants (48.2–56%, *p* < 0.001 vs. 35.7% and 15.2% for WT and Δ*KU70*) (Figure 11b). By contrast, conidia survival of the TRC-grown WT strain was unchanged. 

The inflammatory macrophage response to the *PIG1*-deficient strains was also indirectly evaluated by measuring the level of various pro-inflammatory cytokines released within 12 h post-infection. Although there was a trend toward increasing IL-1β release by M1- and M2-infected macrophages, the IL-18 concentration increased by a factor of 1.9 (*p* < 0.05), 5.0 (*p* < 0.001), and 6.1 (*p* < 0.001) for macrophages infected with the T, M1, and M2 transformants, respectively, relative to the parental strain, (Figure 11c). However, there was no significant change in the level of IL-6, TNFα, IL-12p70, IL-17A, IL-23, or IL-10 released by macrophages infected by any of the mutants (Figure 11c).

## 4. Discussion

The relevance of DHN-melanin in fungal virulence has been increasingly assessed in recent decades for both plant and human pathogens. In addition to protecting fungal structures from environmental stresses, the melanin layer of the conidia cell wall has been recognized in certain genera to be an effective camouflage against the host immune system. In certain phytopathogens in which DHN-melanin has been extensively explored as a key-component of invasion organs, called appressoria [48], melanin biosynthesis involves several enzymes under the regulation of conserved and mostly clustered genes. In particular, in *Magnaporthe oryzae* [31], *Colletotrichum gloeosporioides* [51], *Cochliobolus heterostrophus* [32], and *Alternaria brassicicola* [14], a transcription factor has been identified within the cluster and numerous studies have shown its activating role in melanization, which is subsequently related to virulence. 

As there are only limited data on the intrinsic factors related to *S. apiospermum* pathogenicity, we aimed to assess the role of the transcription factor PIG1 in the biosynthesis of DHN-melanin in *S. apiospermum* conidia. As an ortholog gene encoding the PIG1 transcription factor was identified in the *S. apiospermum* genome, a CRISPR-Cas9-mediated *PIG1* deletion was conducted on two melanized *S. apiospermum* strains, the WT reference 14462 strain and a Δ*KU70* deleted strain derived from the reference strain. Three melanin-deficient recombinants displaying white colonies were obtained, although the complete *PIG1* deletion was obtained only in the two Δ*KU70-*derived mutants. Unexpectedly, the 14462 WT-derived T strain integrated the repair template upstream of *PIG1*. This may be explained by (i) an ectopic insertion in this area or (ii) by a the *HPH* insertion by a micro-homology-end joining mechanism as recently described in *Magnaporthe oryzae* [52]. This mutant T strain was indeed able to grow similarly in the presence of hygromycin as the Δ*KU70*-derived mutants, and the function of PIG1 was apparently hampered, as shown by the absence of melanin in the conidia. However, the increased production of *PIG1* mRNA observed suggests that a gene-silencing mechanism may occurred by transcriptional/post-transcriptional interference [53]. 

To assess the role of DHN-melanin in *S. apiospermum*, some experiments included a control tricyclazole (TRC)-treated WT strain acting on specific reductases from the DHN-melanin biosynthesis. Indeed, TRC-treated colonies appeared lightly pigmented due to the accumulation of melanin shunt intermediates. Thus, even if there were some slight differences with albino strains, this condition generated melanin-lacking spores. This comparison allowed us to confirm the involvement of the melanin in some observed phenotypes, and to reveal additional melanin-independent phenotypes, possibly related to PIG loss. Besides, we analyzed the global transcriptome of M1 relative to its parental strain Δ*KU70* to identify differentially expressed genes (DEGs) that could explain the melanin-independent phenotypes observed.

For the first time, the phenotype of melanin-lacking spores of *S. apiospermum* was deeply explored, emphasizing the major structural role of DHN-melanin in the conidia cell wall. It is commonly thought that DHN-melanin plays a role of “fungal armor” considering its external location in the cell wall. This has been supported by the increase of polysaccharide exposure in conidia from the three transformants together with TRC-treated strain. The structural disorganization may be associated with the reduced cell wall thickness highlighted in T, M1 and M2 conidia. These results are in line with a previous report revealing the drastic decrease in the cell wall thickness of *Alternaria alternata* deficient conidia, following pyroquilon-mediated DHN-melanin inhibition [54]. Similarly, the cell wall of TRC-treated hyphae from another phytopathogen fungus *Gaeumannomyces graminis* (*Magnaporthaceae* family) was thinner than the untreated strain [55]. 

Moreover, several unexpected phenotypes were observed in the *PIG1* recombinants that cannot definitely be the consequence of melanin-deficiency but result from the absence of functional PIG1. 

The conidia morphology of the three recombinants appeared to be equally affected, showing aberrant septate and large conidia, contrasting with the regular and ovoid unicellular spores released by the parental strains and the tricyclazole-treated strain. No change in conidia shape has previously been associated with melanin deficiency due to the specific disruption of genes encoding biosynthetic enzymes, such as polyketide synthase or scytalone dehydratase. This observation has also never been reported in mutants with the deletion of *PIG1* orthologs from fungi producing monocellular conidia [51,52]. By contrast, in some genera that normally produce pluricellular conidia, a reduced number of conidia septations has previously been reported, such as for conidia from *CMRA*-deleted *Alternaria alternata* [56] or tricyclazole-exposed *Bipolaris* [57]. 

The complexity of the cell-wall architecture, which is still partially unresolved, did not allow us to definitively identify the differentially expressed gene(s) responsible for the presence of compartmentalized conidia. Although the process or functions could not be determined for all DEGs, several important enzymes dedicated to processes associated with cell-wall metabolism were overexpressed in M1 relative to WT, such as a neutral/alkaline ceramidase. The fungal cell wall is a dynamic and complex structure in which polysaccharides, oligosaccharides, lipids, and proteins are intertwined [24]. For the first time, the transcriptomic analysis of a Δ*PIG1* fungal strain was performed. Interestingly, a significant number of genes encoding hydrolases, glycosyltransferases, and other enzymes involved in carbohydrate metabolic processes were differentially regulated in the M1 recombinant. In *Scedosporium*, glycosphingolipids are components of the plasma membrane, the cell wall, and extracellular vesicles involved in fungal growth and virulence [26,58]. In particular, glucosylceramides (GlcCer) are major neutral sphingolipids bound to the fungal cell surface, characterized by a fatty-acid chain bound to a sphingosine and a sugar unit [59] and highly conserved among pathogenic fungi, such as *Candida albicans*, *Cryptococcus neoformans*, and *Aspergillus nidulans* [60,61,62]. The crucial role of GlcCer in fungal physiology, i.e., in cell division, growth, alkaline tolerance, the promotion of virulence, and the modulation of the host immune response [63], and their unique structure compared to the components of mammalian cells, make them interesting targets for new antifungal strategies [25,64,65]. Although there are some data on the essential role of GlcCer in fungal homeostasis, there is no data on the specific role of the neutral and alkaline ceramidases that regulate ceramide and sphingosine levels, *a fortiori* in *S. apiospermum*. Thus, the overexpression of a neutral/alkaline ceramidase resulting from *PIG1* inactivation in the Δ*PIG1* recombinant may hinder normal physiology, but this hypothesis is yet to be specifically addressed. Finally, little is known about the function of conidiation specific protein 6 (CON6), which was downregulated in the two Δ*PIG1* recombinants. Initially described in *Neurospora crassa*, a filamentous fungus used as a model for studies on developmental processes, the transcription of this regulatory gene increases during conidiation and following exposure of vegetative mycelia to light [66,67]. However, its exact function and association with the phenotype of the *PIG1* mutants are still unknown. 

In addition, PIG1 is involved in the protection of conidia heat stress as revealed by the higher susceptibility of Δ*PIG1* disruptants to heat relative to their parental strains. Similar observations were previously made in *Verticillium dahliae* with a deletion of *CMR1*, an ortholog of *PIG1,* when exposed to heat stress [23,68]. 

In recent years, our understanding of host-fungi interactions has led to a great interest in deciphering the role of surface cell-wall components in fungal virulence, with specific attention on DHN-melanin. In *Scedosporium* species, polysaccharides and peptidopolysaccharides are especially relevant for the architecture of the conidia cell wall and several are immunologically active, with great potential as regulators of pathogenesis and the host immune response. For example, a significant reduction in both phagocytosis and killing of *S. apiospermum* conidia was demonstrated using anti-peptidoramnomannan mAbs in murine macrophage-like cells relative to control conidia [69]. Here, the removal of DHN-melanin resulted in equally higher exposure of underlying cell-wall polysaccharides in the three mutants and the TRC-treated WT strain, potentially increasing the availability of surface antigenic patterns and conidia interaction with phagocytic cells. Thus, we assessed the in vitro response of human macrophages to infection by *PIG1* recombinants relative to their parental strains. However, the uptake (adherence) of T, WT-TRC, M1 and M2 conidia by macrophages relative to their parental strain let to heterogeneous findings between WT and Δ*KU70*-derived strains, consistent with the contradictory results of previous studies conducted on melanin-deficient fungi [70,71,72]. Then, we showed that the conidial killing of the three melanin-deficient recombinants was equally enhanced, which unexpectedly contrasted with the unchanged survival of WT-TRC conidia. Together with the enhanced macrophage release of IL-18 following infection with T, M1 M2 but not with WT-TRC conidia, these results suggest the role of PIG1 in conidia survival upon macrophage interaction, rather than the direct involvement of melanin.

Other phenotypes were described, without establishing if they were related to the loss of melanin or to the absence of functional PIG1.

We showed a reduced growth of *PIG1* recombinants incubated with cell wall stressors (Congo Red, SDS) and oxidative chemicals, in particular cumene. Such increased vulnerability may result from a combination of several mechanisms. First, the radical structure of DHN-melanin provides free radical scavenging properties, which could explain why, in addition to the loss of cell-wall integrity, the three melanin-deficient mutants were less resistant to oxidizing molecules. Another important point is that fungi, *Scedosporium* in particular, possess a considerable enzymatic arsenal capable of detoxifying numerous toxic compounds, especially those that induce oxidative stress [73,74]. In the M1 recombinant we highlighted the dysregulation of various enzymes that play a role in oxidoreduction processes, such as the protection against oxidative stress, for example, through glutathione metabolism. It is therefore likely that certain overexpressed enzymes, such as thioredoxin reductase, shown in the M1 disruptant, play a role in the detoxification of menadione-generated superoxides. 

DHN-melanin has been commonly associated with fungal protection against stress that triggers cell damage, such as electromagnetic radiation and oxidants [12,75,76,77]. This is what we observed in the Δ*KU70* strain exposed to UV-C light relative to both the Δ*KU70*-derived M1 and M2 recombinants. By contrast, the WT strain showed much lower UV resistance than its derived Δ*KU70* strain and paradoxically, its melanin-deficient derived recombinant tended to be more resistant to UV light. These results prevent consistent conclusions to be drawn regarding the role of PIG1 orthologs and DHN-melanin in *S. apiospermum* protection against UV exposure, in line with previous contradictory findings varying according to fungal models [14,29,51]. 

The melanization of the fungal cell wall has also previously been linked to antifungal resistance, especially for *C. neoformans*, *Histoplasma capsulatum* [21], and *Exophiala dermatitidis* against AmB [23] or *Madurella mycetomatis* against azoles [78]. Here, the susceptibility to triazoles and anidulafungin was unchanged by *PIG1* deletion in *S. apiospermum,* and the M1 and M2 disruptants sporadically recovered AmB susceptibility, relative to the parental Δ*KU70*. Thus, we were not able to draw any conclusions about the role of melanin in *S. apiospermum* antifungal susceptibility. Of note, the mechanism explaining the lower susceptibility of the Δ*KU70* strain to AmB than the 14462 WT strain is still unclear. Nonetheless, independently of DHN-melanin, the restored susceptibility of the Δ*KU70*-derived recombinants to AmB may be linked to the downregulation of certain key-antioxidative enzymes, such as catalases, as shown by the transcriptomic analysis. Indeed, the AmB resistance of *Scedosporium*/*Lomentospora* has been recently shown to be associated with the ability to thwart the cellular redox imbalance triggered by AmB through potent activity of antioxidative enzymes [79]. 

Finally, the heterogeneous phenotype between the two melanized parental strains observed for some assays, in particular those dedicated to evaluating UV resistance, biofilm formation and antifungal susceptibility, could be possibly related to the higher melanin content in Δ*KU70* conidia, as well as the absence of the KU70 protein itself [80,81]. Indeed, although the benefit of *KU70* deletion is widely recognized to facilitate genetic transformation in fungi, the loss of this component of the nonhomologous end-joining pathway involved in DNA double-strand break repair could also have an impact on other physiological processes, which has never been specifically explored.

## 5. Conclusions

Overall, we demonstrate that the transcription factor *PIG1* regulates the DHN-melanin biosynthetic pathway in *S. apiospermum* through the construction of *PIG1*-deleted strains, which displayed phenotypic features similar to previously described *PKS*-disrupted fungi. Supported by the transcriptomic analysis, this study highlights the pleiotropic action of *PIG1* in *S. apiospermum* beyond melanin regulation, including on the cell-wall architecture and enzymatic pathways. Although, we were unable to determine whether certain phenotypic observations were attributed directly to the loss of the melanin layer or due to a collateral effect of PIG1 loss of function, we provide evidence for the involvement of PIG1/melanin in the protection of conidia against environmental damages and macrophage killing, and possibly in the pathogenicity of *Scedosporium*. 

This study provides new insights on the melanization process of *S. apiospermum* and paves the way for further research on DHN-melanin as a potential therapeutic target in a fungus with low susceptibility to most current antifungal options. 

## Figures and Tables

**Table 1:** Minimum inhibitory concentration (MIC) determined by the broth microdilution method. MICs determined after incubation for 72 h at 37 °C as the lowest concentration of antifungal to result in a growth reduction of 90% (amphotericin B) or 50% (triazoles) relative to the growth control in antifungal-free RPMI. Growth was assessed by measuring the optical density at 405 nm. Significant differences in the MIC are shown in bold. T: transformant derived from the WT strain; M1 and M2: transformants derived from the Δ*KU70* strain.

Antifungal	Minimum Inhibitory Concentration (Mean ± SD, µg/mL)				
	WT	T	Δ*KU70*	M1	M2
Amphotericin B	0.58 ± 0.38	0.50 ± 0.43	**>8**	**0.58 ± 0.38**	**0.83 ± 0.29**
Voriconazole	0.34 ± 0.19	1.37 ± 0.75	1.37 ± 0.75	0.87 ± 0.25	0.87 ± 0.25
Itraconazole	0.75 ± 0.43	0.83 ± 0.29	1.33 ± 0.58	0.83 ± 0.29	1.33 ± 0.58
Posaconazole	0.42 ± 0.14	1.33 ± 0.58	0.83 ± 0.29	1.33 ± 0.58	1.0 ± 0
Isavuconazole	6.67 ± 2.3	8 ± 0	8 ± 0	4 ± 0	5.3 ± 2.3
Anidulafungin	>8	>8	>8	>8	>8

**Table 2:** qPCR validation of RNA-seq results on selected genes.

Gene CDS ID	Description	M1		
		RNA-seq		qPCR
		Log_2_FC	*p*	Log_2_FC
SAPIO_CDS8655	*PIG1*	**−3.7890**	4.62 × 10^−12^	−15.09
SAPIO_CDS5047	Scytalone deshydratase (*SCD*)	**−6.1180**	1.08 × 10^−36^	−4.825
SAPIO_CDS8657	Tetrahydroxynaphthalene reductase (*THNR*)	−1.7735	0.2442	−2.533
SAPIO_CDS8658	Conidial yellow pigment biosynthesis polyketide synthase (*PKS*)	**−1.7835**	0.0011	−2.133
SAPIO_CDS8659	iron transport multicopper oxidase FET3 (ABR1)	−0.8088	0.1608	−0.881
SAPIO_CDS6958	Conidiation specific protein-6 (*CON6)*	**−3.1829**	0.0298	−3.343
SAPIO_CDS1830	Thioredoxin reductase	1.5835	0.2752	0.5800
SAPIO_CDS8079	Peptidase C2, calpain family	**−5.2099**	1.60 × 10^−10^	−5.305

Log_2_FC: mean values from four independent biological replicates. Statistically significant differential gene expression values (log_2_FC) are shown in bold. M1: transformant derived from the Δ*KU70* strain.

## Data Availability

The data presented in this study are available on request from the corresponding author.

## Supplementary Materials

The following supporting information can be downloaded at: https://www.mdpi.com/article/10.3390/jof9020134/s1, Figure S1: Fluorescence labelling of conidia surface carbohydrates with FITC-conjugated lectins; Table S1: Nucleotide sequence of constructs and primers used in the study; Table S2: Summary of RNA-Seq quality data of M1 and its Δ*KU70* parental strain. Four biological repeats were performed for each isolate.
