# Supplementary material for: Deciphering the Role of PIG1 and DHN-Melanin in Scedosporium apiospermum Conidia
Source: J Fungi (Basel). 2023 Jan 18;9(2):134. doi: 10.3390/jof9020134 (PMC9965090; doi:10.3390/jof9020134)
Supplement: Supplementary file 1 [file jof-09-00134-s001.zip › jof-2127933-supplementary.pdf]

**Supplementary Materials:**

**Figure S1.** Fluorescence labelling of conidia surface carbohydrates with FITC-conjugated lectins.
**Table S1.** Nucleotide sequence of constructs and primers used in the study
**Table S2**. Summary of RNA-Seq quality data of M1 and its Δ*KU70* parental strain. Four biological repeats were performed for each isolate.

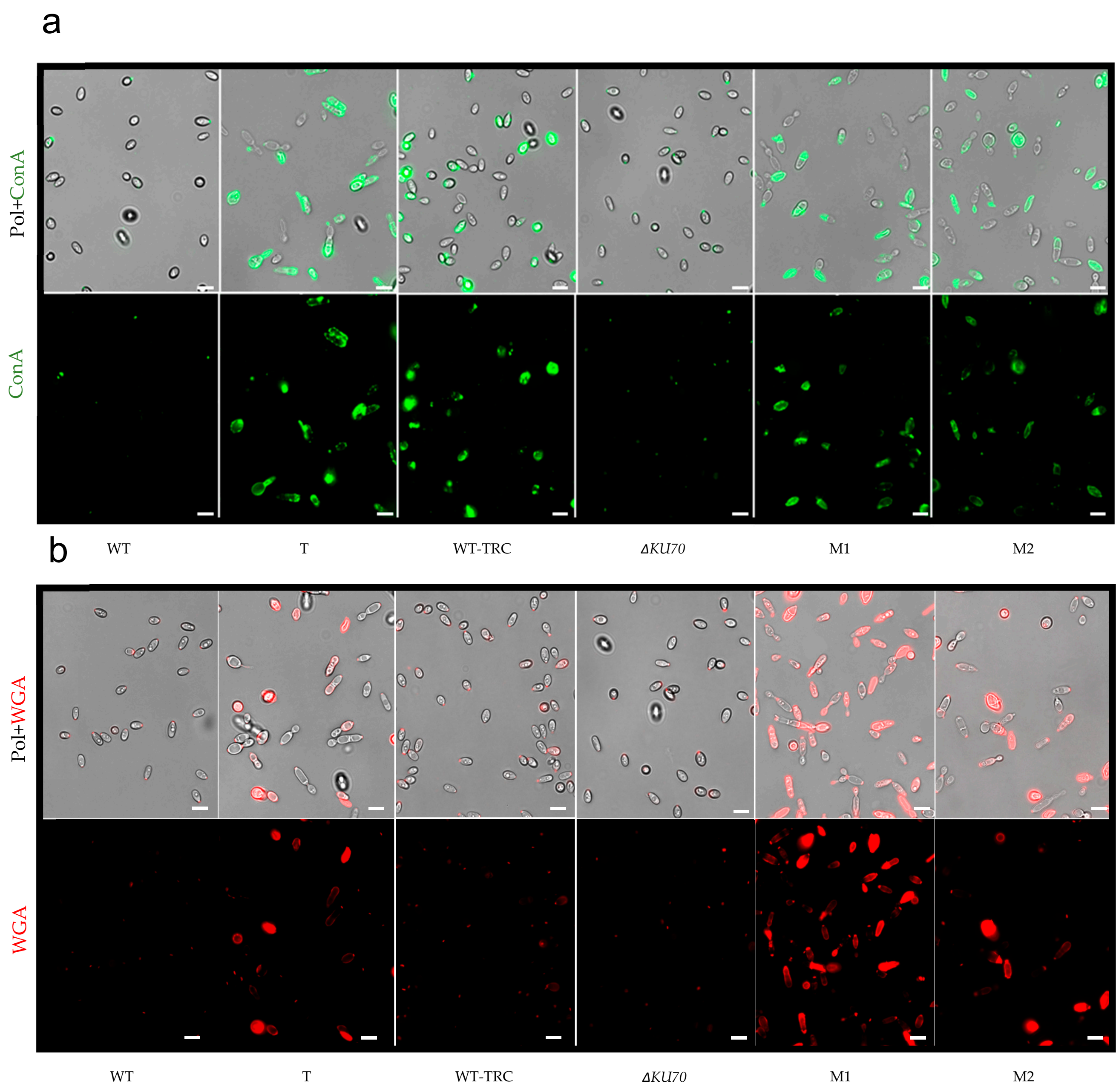


**Figure S1**. Fluorescence labelling of conidia surface carbohydrates with FITC-conjugated lectins. Representative images from fluorescence acquisition of conidia stained with Concanavalin A (**a**) or WGA (**b**), which bind to mannosyl and glucosyl residues and N-acetylglucosamine residues, respectively. Image acquired using an Olympus IX71 widefield inverted fluorescence microscope using a 20× magnification lens. Scale bar: 10μm.

**Table S1.** Nucleotide sequence of constructs and primers used in the study

| Gene target | Sequence (5′-3′) | Application |
|---|---|---|
| crRNA protospacer (*PIG1*) | GACGGCCAAGCAACTTGTGG | CrispR-Cas9 construction |
| tracrRNA | GTTTTAGAGCTAGAAATAGCAAGTT AAAATAAGGCTAGTCCGTTATCAAC TTGAAAAAGTGGCA CCGAGTCGGTGCT | |
| Up-stream and down-stream region of *PIG1* (SAPIO_CDS8655) | F- TGTGACACAAGGCTTATACC<br>R- TACGAGCGAGCCTTTTGTCT | PCR (verification of *PIG1* deletion) |
| *FIS1* (SAPIO_CDS7496) | F-GATCAGTTCGAGAAGGAGGG<br>R- GGAAGATGTCGGAGAGGAGA | RT-qPCR (RNA-seq validation) |
| *UBC6* Ubiquitin-conjugating enzyme E2 J2 (SAPIO_CDS4581) | F-GAGAACCCGCCACCATACA<br>R-CAATACTGGCCGCCATGGTA | |
| *PIG1* (SAPIO_CDS8655) | F-TTTGGGTGCTTGGAGTGGGGATAA<br>R-GCCGTATCGTGGAACAAGCTCAAT | |
| Tetrahydroxynaphthalene reductase (SAPIO_CDS8657) | F-GGGCAAGGTCGCTGTAGTCA<br>R-TGGGGTTGGTGCCGAAAGAC | |
| Polyketide synthase (SAPIO_CDS8658) | F- AGAAGTCGATGGCTTGACGAGG<br>R- GGCAGTCCAATCTTCAGGCTCT | |
| Iron transport multicopper oxidase (abr1, SAPIO_CDS8659) | F-CCACCGGGCGAAAGTATTGTGT<br>R-CGTCGGGGTATTGTGCCATGTT | |
| Scytalone deshydratase (SAPIO_CDS5047) | F- CACCCTCAAGACGCAGCACT<br>R- CCCGTCCACCCTCCTATACCAA | |
| Peptidase C2, calpain family (SAPIO_CDS8079) | F-AGGTTTGCCTCGTGCTGCAT<br>R-ATCATGCGCCTTTTCATGCCCT | |
| Conidiation specific protein-6 (SAPIO_CDS6958) | F-AGATTGACACCTCTCCTAGCGCC<br>R-TGCTTCCCGTTTGGCCTCCT | |
| Thioredoxin reductase (SAPIO_CDS1830) | F-GACGTGAATCCTGCCACTTT<br>R-ACCGAGAATGTCACCCTGTA | |

F: forward primer, R: reverse primer

**Table S2**. Summary of RNA-Seq quality data of M1 and its Δ*KU70* parental strain. Four biological repeats were performed for each isolate.

| Index | **Δ*KU70*** | | | | **M1** | | | |
|---|---|---|---|---|---|---|---|---|
| | 1 | 2 | 3 | 4 | 1 | 2 | 3 | 4 |
| Total reads (millions) | 17.6 | 20.6 | 20.8 | 20.7 | 19.8 | 12.7 | 20.6 | 17.3 |
| Total assigned reads (millions) | 11.7 | 12.3 | 15.0 | 14.6 | 10.4 | 9.4 | 14.6 | 11.2 |
| % assigned reads | 66.2 | 59.8 | 71.8 | 70.3 | 70.7 | 74.1 | 70.4 | 64.7 |
| Q30% | 95.7 | 95.6 | 95.4 | 95.5 | 95.8 | 95.5 | 95.7 | 95.7 |
| GC% | 53.0 | 52.0 | 53.7 | 53.5 | 53.3 | 55.0 | 53.5 | 52.9 |

Q30%: proportion of reads with a quality value > 30 after filtering. GC%: proportion of guanidine and cytosine nucleotides among total nucleotides after filtering.
